# The chain mediating effect of empathy and communication ability on emotional intelligence and caring ability of nursing students

**DOI:** 10.3389/fpsyg.2023.1339194

**Published:** 2024-01-08

**Authors:** Yujie Yang, Chang Wang

**Affiliations:** ^1^International School of Nursing, Huangshan Vocational and Technical College, Huangshan, Anhui, China; ^2^Department of Orthopaedics, Huangshan Xinchen Hospital, Huangshan, Anhui, China

**Keywords:** nursing student, caring ability, emotional intelligence, empathy, communication ability, mediation analysis

## Abstract

**Background:**

The implementation of humanistic care is conducive to providing high quality nursing, improving patient satisfaction and establishing harmonious nursery-patient relationship. Current researchs show that humanistic caring ability is affected by emotional intelligence, empathy, interpersonal communication. But the exact relationship and internal mechanism of such factors have not been fully understood.

**Objective:**

To explore the multiple mediating effect of empathy and communication ability on nursing students’ emotional intelligence and caring ability.

**Methods:**

A case study was conducted by examining a sample of 1,165 nursing students from a junior college in Anhui, China. The multidimensional scales, and a self-designed demographic characteristics questionnaire were utilized. Path relationships and mutual effects were tested using structural equation modeling (SEM).

**Results:**

Emotional intelligence, empathy, and communication ability were found to positively affect nursing students’ caring ability, as well as positive interrelationships with one another (standardized estimate = 0.312–0.584, *p* < 0.001). Communication ability and empathy play an important role in mediating the association between emotional intelligence and caring ability, and the effect sizes are 0.169 and 0.121, respectively, while the effect value of empathy and communication ability in emotional intelligence and caring ability was 0.034, which showed partial mediation of the association. The indirect effect of the structural equation was 77.14%.

**Conclusion:**

The chain mediating role of empathy and communication ability in emotional intelligence and caring ability is explored, which not only enrichis previous studies, but also reveals the mechanism of emotional intelligence’s influence on caring ability. It is essential to continuously improve nursing students’ caring ability. From the perspective of nursing educators, they need to develop targeted approaches to help the nursing student improve their emotional intelligence, empathy, and communication skills, further enhancing their caring ability.

## 1 Introduction

Humanistic care refers to the capability of nursing professionals to attentively listen to patients’ desires and needs, effectively communicate with them, empathize with their emotional experiences, and recognize the intrinsic value of life, all of which facilitate the development of therapeutic relationships (Rogers, 1981). In such care, emphasis is placed on caring for the “whole person,” encompassing patients’ pphysical, mental, cognitive, emotional, spiritual, and social conditions, rather than focusing narrowly on the physical elements of the illness. The ability of medical staff to provide humanistic care was clearly characterized in terms of professional performance and patients’ quality of life (Cheng et al., 2016; Aupia et al., 2018). Since the era of Florence Nightingale, nursing has been a profession that requires service, caring, and compassion. Since the implementation of the “High-Quality Nursing Service” program in 2010, China has made it even more clear that clinical practitioners are required to incorporate a “patient-centered” service philosophy and care concept into their daily routines. Further, the “Healthy China 2030″ planning framework was proposed in 2016, which deeply clarified the direction of and support for nursing promotion. To provide high-quality care for patients, medical professionals should foster the values of warmth, individual care, and compassion in their clinical work.

Despite the theoretical and policy emphasis on the essentiality of nursing promotion, the current caring level of nursing remains at a relatively low level (Cheng et al., 2016; Xu et al., 2021). As nursing students represent a crucial source of future clinical care providers, it is increasingly important to prioritize and foster their ability to provide humanistic care. To effectively improve nursing students’ caring ability, this study will examine the inherent qualities and individual factors that can be modified positively.

## 2 Literature review and research hypothesis

### 2.1 Caring ability

Caring ability refers to nurses’ ability to care, support, and assist patients to obtain comprehensive physical and mental health and harmonious life, which is the core ability of nurses to carry out high-quality nursing services. The nurses with high caring ability have more efficient and comprehensive performance in clinical practice and nursing operations, which can reduce workload, increase patient satisfaction, and further contribute to the derivation of a harmonious environment (Létourneau et al., 2021; Zhong et al., 2021).

Caring ability is not innate, as a special ability, the cultivation of humanistic caring ability not only needs their own continuous learning and experience accumulation, but also needs the training education acquired. Nursing in China has gained greater progress on the path of humanistic care in the past few decades. However, in the face of increasing patient needs, especially spiritual needs such as caring and respect, the education and practice of humanistic care has been accelerated and insufficient, which has further hindered the clinical caring quality (Xu et al., 2021; Hu et al., 2022). Therefore, nursing educators and managers should incline more focus on exploring and practicing the cultivation methods. Previous studies have shown that clinical practice, high-fidelity simulations and Narrative pedagogy are conducive to improving humanistic caring ability (Kuo et al., 2021; Létourneau et al., 2022; Xue et al., 2023). Current research on influencing factors show that emotional intelligence, empathy, and interpersonal communication influence humanistic caring ability (Jian et al., 2022; Ma et al., 2022). This study will verify and explore the relationship and mechanism of the influencing factors, and provide reference for cultivating humanistic caring ability.

### 2.2 Emotional intelligence

Emotional intelligence refers to individuals’ ability to regulate, comprehend, and perceive emotions of those around them, as well as to solve problems through emotional means (Mayer et al., 2008), which includes the ability to accurately evaluate their own and others’ emotions, appropriately express emotions, and adjust emotions adaptively (Wessel et al., 2008). The prior research observed a significant positive correlation between emotional intelligence and caring ability (Wang et al., 2020). The higher the nursing student’s level of emotional intelligence, the better them can observe the emotional changes of others. In addition, it empowers nursing students to retrieve and perceive changes in the emotions, including patients. Such students can accurately identify emotional fluctuations in patients, comprehend their physical and psychological distress, address their needs, and provide them with humanistic care. Therefore, emotional intelligence can improve caring ability. This study puts forward the *hypothesis 1: There is a remarkably positive correlation between emotional intelligence and caring ability.*

### 2.3 Empathy

Empathy is individuals’ “ability” to comprehend the emotional state. It is often portrayed as individuals’ ability to empathize with the other person (Ioannidou and Konstantikaki, 2008). Previous research has shown that more empathic behavior tends to occur in people with higher emotional intelligence (Silva and Toledo Júnior, 2021; Wan et al., 2023). It is clear that individuals who have strong perception, identification and control of their own emotions are equally able to gain mastery over others. Emotionally intelligent people tend to empathize with others by understanding the deeper reasons for their somatic expressions. Therefore, the development of empathy also drives the development of an individual’s emotional intelligence (Xiang et al., 2022). Therefore, this paper proposes *hypothesis 2: There is a remarkably positive correlation between emotional intelligence and empathy.*

In previous studies, scholars state a positive association between empathy and caring ability (Korkmaz Doğdu et al., 2022; Liu et al., 2023). Emotional intelligence can assist nurses in empathizing with the patients they are attending to, enabling them to accurately perceive patients’ emotions from their perspective, understand their needs and emotions, and provide care based on patients’ perspectives, thereby enhancing their humanistic care abilities. Therefore, *hypothesis 3 is proposed: There is a remarkably positive correlation between empathy and caring ability.*

### 2.4 Communication ability

Communication ability has long been considered an essential quality for nurses, which is the foundation of quality nursing practice (Xie et al., 2013). The existing studies claim a positive relationship between communication and emotional intelligence (Li et al., 2021b). Individuals with better emotional intelligence can comprehend emotions and exhibit discernment in interpersonal connections, which can aid in improving communication skills and fostering harmonious relationships (Petrovici and Dobrescu, 2014). Therefore, *hypothesis 4 is proposed: There is a remarkably positive correlation between emotional intelligence and communication ability.*

Previous studies have shown that interpersonal communication ability directly affects caring ability (Li et al., 2022). Individuals with good communication skills can apply their communication skills to their patients more easily and quickly and meet the psychological needs of patients. Nurses convey sympathy and support to patients through communication, which is an important way to implement humanistic care. Therefore, We propose *hypothesis 5: There is a remarkably positive correlation between communication ability and caring ability.*

Previous studies have shown that empathy is a facilitator for effective communication (Giménez-Espert et al., 2019; Cannity et al., 2021). Individuals with the ability to empathize can accurately grasp the key points that patients hope to be cared for, and can always make patients feel happy both physically and mentally during the communication process. Individuals with empathy have an advantage in perceiving the emotional fluctuations of patients and are able to put themselves in the patient’s shoes and communicate their inner feelings. Therefore, *hypothesis 6 is proposed: There is a remarkably positive correlation between empathy and communication ability.*

The empathy and communication ability may have some mediating effect between caring ability and emotional intelligence (Wang et al., 2020; Li et al., 2021a; Lina et al., 2022). However, the exact mediating relationship and internal mechanism of such factors have not been fully understood. Based on previous studies and the hypotheses mentioned above, the mediating effect hypotheses are proposed.

*Hypothesis 7*. Empathy mediates the effect of emotional intelligence on caring ability.

*Hypothesis 8*. Communication ability mediates the effect of emotional intelligence on caring ability.

*Hypothesis 9*. Empathy and communication ability mediate the effect of emotional intelligence on caring ability.

**Figure 1:**
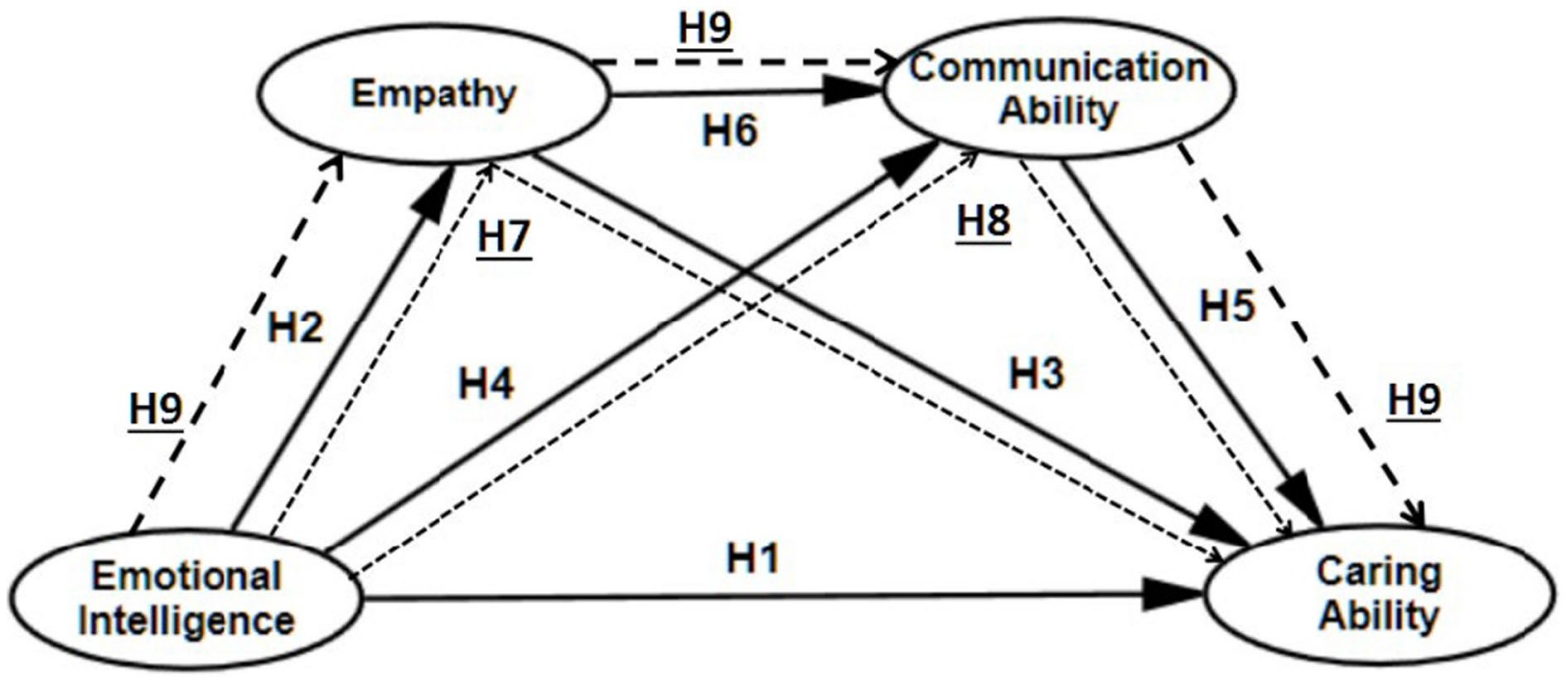
Hypothesized model.

On the basis of existing research, a theoretical model of the caring ability was developed (Figure 1) and proposed hypotheses. The aim was to investigate the mediating impact of emotional intelligence on caring ability and provide evidence for nursing educators to take targeted approaches to enhance nursing students’ caring ability.

## 3 Methods

### 3.1 Research setting and participants

The ethical review board of the authors’ college approved the work. From October to November 2022, the nursing students from a college were selected in Anhui Province, China, using the convenience sampling method. Before the investigation, researchers clarified the importance of this study and got informed consent from these students, informing them that the investigation followed the principle of voluntariness and all data were for research purposes only. Students meeting the following criteria could participate in the study: the participants had to be nursing students, had to comprehend the research purpose and participate voluntarily. Eventually, 1,290 students completed the survey, and 1,165 valid questionnaires were collected, with the effective rate reaching 90.31%.

### 3.2 Measures

#### 3.2.1 Demographic characteristics questionnaire

The questionnaire included demographic information such as age, gender, family location, and whether the participant was an only child or a class leader, among other items.

#### 3.2.2 Caring ability inventory (CAI)

In 1990, Nkongho first developed the Caring Ability Inventory, which is a widely used research approach. In the Chinese version translated by Xu and Liu, this concept contains three aspects: a courage aspect with 13 items, a cognitive aspect with 14 items, and a patience aspect with 10 items. The inventory consists of 37 items, and responses are given using a 7-point Likert scale. The total score is 37–259 points, and higher scores indicate better caring ability. The Cronbach’s α was 0.967, and the dimension was 0.923 ~ 0.970.

#### 3.2.3 Emotional intelligence scale (EIS)

The Emotional Intelligence Scale was first proposed as a self-report scale by Schutte et al. in 1998. The Chinese version (Wang, 2002) contains four aspects: (1) evaluation of self-emotions with 12 items, (2) adjustment of self-emotions with 8 items, (3) understanding of others’ emotions with 6 items, and (4) employment of emotions with 7 items. It uses a 5-point Likert scale, in which 1 point indicates complete inconsistence and 5 points indicate full compliance, and higher scores indicate greater emotional intelligence. The Cronbach’s α of the scale was 0.966, and the dimension was 0.865 ~ 0.931.

#### 3.2.4 Interpersonal reactivity index (IRI)

The Interpersonal Reactivity Index, initiated by Davis in 1983, is a method used to for measure empathy capabilities. It was transformed into Chinese, with some adjustments from the original 28 items to the current 22 items (Guan and Qian, 2014). The scale has four aspects: fantasy, perspective taking, personal distress, and empathy concern, including 6 items, 5 items, 5 items, and 6 items, respectively. Higher scores indicate greater empathy. In the present study, the Cronbach’s α was 0.953, and the dimension was 0.847 ~ 0.876.

#### 3.2.5 Supportive communicative scale (SCS)

The Supportive Communicative Scale is an approach for evaluating interpersonal communication skills and it was initially proposed by Whetten and Cameron in 1998. The Chinese version translated by scholars Gao, Yuan, Lei, and Fan in 2009 consisted of three aspects: coaching and counseling, the supply of effective negative feedback, and supportive communication, containing 3 items, 6 items, and 11 items, respectively. It used a 5-point Likert scale, in which 1 point represent complete inconsistence and 5 points represent full compliance. The higher scores represent better communication skills. The scale had Cronbach’s α of 0.940, and the dimension was 0.767 ~ 0.912 in the present study.

### 3.3 Data analyses

For descriptive statistical analysis and Pearson correlation analysis, this study adopted SPSS statistics version 23.0. To test the hypothesized model, SEM was adopted. Jöreskog and Sörbom (1982) claimed that in the hypothesized model, researchers can use SEM to conduct a maximum likelihood estimation of the whole system and use the data to analyze variables. This study also examined the hypothesized model using the two-step approach proposed (Anderson and Gerbing, 1888). To start with, this study ascertained the measurement using model confirmatory factor analysis (CFA). Next, researchers analyzed the path and fit coefficients of the model through SEM analysis, analyzing various indicators such as degrees of freedom (df), the chi-square (χ^2^) value, the chi-square degrees of freedom (χ^2^/df), comparative fix index (CFI), standardized root mean square residual (SRMR), Tucker-Lewis index (TLI), and the root mean square error of approximation (RMSEA). Good fit is indicated by χ^2^/df < 3, CFI > 0.95, TLI > 0.95, SRMR <0.06, and RMSEA <0.06, and χ^2^/df < 5, CFI > 0.90, TLI > 0.90, SRMR <0.08, and RMSEA <0.08 were acceptable (Hu and Bentler, 1999).

## 4 Results

### 4.1 Demographic

Regarding the demographic features, the mean age of the 1,165 participants was 18.056 ± 1.526 years, 118 (83.9%) were male, 977 (16.1%) were female, and 379 (32.5%) reported being the only child in their family. For family location, 231 people (19.8%) came from cities, 345 people (29.6%) came from towns, and 589 people (50.6%) came from rural areas. Of the participants, 484 (41.5%) had been class leaders. For the experience of caring, 605 (51.9%) of the students had the caring experience.

### 4.2 Preliminary analyses

#### 4.2.1 Variable correlation analysis

The associations and reliability are manifested in Table 1. The Cronbach’s α of four scales was 0.940 ~ 0.967. Through Pearson correlation analysis, the authors observed a positive correlation between emotional intelligence and empathy (*r* = 0.290), communication ability (*r* = 0.420), and caring ability (*r* = 0.329). Both empathy and communication ability positively influence caring ability (*r* = 0.396; *r* = 0.482, respectively), and empathy positively influences communication Ability (*r* = 0.337). There was a statistical significance in the differences (*p* < 0.01).

**Table 1:** Reliability and Pearson correlation of variables.

	Alpha	AVE	Emotional intelligence	Empathy	Communication ability	Caring ability
Emotional Intelligence	0.966	0.744	**0.863**			
Empathy	0.953	0.749	0.290^**^	**0.865**		
Communication Ability	0.940	0.693	0.420^**^	0.337^**^	**0.832**	
Caring Ability	0.967	0.558	0.329^**^	0.396^**^	0.482^**^	**0.747**

(1) ^*^p < 0.05; ^**^p < 0.01 (two-tailed); *N* = 1,165. (2) Square root of AVE in bold on diagonals.

#### 4.2.2 Reliability and validity tests

The confirmatory factor analysis (CFA) analysis was employed to evaluate the various accuracies of the four constructs (see Table 2) in the hypothetical model. In this model, the measured variables had varied Cronbach’s α coefficients, falling in the interval of 0.767 ~ 0.970, the squared multiple correlation (SMC) was between 0.477 ~ 0.792, and the factor loadings were between 0.691 and 0.890. The CR of these constructs was 0.791–0.923, and their AVE ranged from 0.558 to 0.750, which is consistent with the standards developed by Fornell and Larcker (1981) and Hair et al. (2006): (1) CR bigger than 0.6; (2) factor loadings higher than 0.5; (3) AVE greater than 0.5; and (4) SMC greater than 0.5. Although the cognitive of squared multiple correlation is slightly lower than 0.5, it still falls within an acceptable range. All other constructs met the required standards, indicating that the four constructs had consistency reliability and convergent validity. Furthermore, we can also conclude from Table 1 that the square roots of the AVE of each construct were greater than their estimated intercorrelations, which preliminarily proved the assertion of Hair et al. (2006).

**Table 2:** Parameter Significance, Reliability and Convergence Effectiveness of Emotional Intelligence, Empathy, Communication Ability and Caring Ability (*n* = 1,165).

Variable	Indicator	Parameter significance estimation	Factor loading	Alpha	SMC	CR	AVE	Alpha			
		Unstd.	S.E.	*t*-value	*p*						
Emotional Intelligence	Appraisal of Own Emotions	1.000				0.870	0.931	0.757	0.921	0.744	0.891
	Regulation of Own Emotions	0.675	0.017	39.583	^***^	0.876	0.904	0.767			
	Understanding of others’ emotions	0.477	0.013	37.160	^***^	0.844	0.856	0.712			
	Utilization of Emotions	0.564	0.015	38.297	^***^	0.859	0.888	0.738			
Empathy	Perspective Taking	1.000				0.868	0.847	0.753	0.923	0.750	0.953
	Fantasy	1.279	0.031	40.782	^***^	0.890	0.876	0.792			
	Empathy concern	1.148	0.030	38.884	^***^	0.865	0.872	0.748			
	Personal distress	1.023	0.028	36.892	^***^	0.840	0.861	0.706			
Communication ability	Coaching and Counseling	1.000				0.713	0.767	0.508	0.870	0.693	0.799
	Providing effective negative feedbacks	2.231	0.082	27.130	^***^	0.884	0.854	0.781			
	Supportive communication	3.475	0.128	27.134	^***^	0.889	0.912	0.790			
Caring ability	Cognitive	1.000				0.691	0.949	0.477	0.791	0.558	0.766
	Courage	1.363	0.069	19.821	^***^	0.795	0.970	0.632			
	Patience	0.763	0.038	19.879	^***^	0.752	0.923	0.566			

Unstd. means unstandardized regression, S.E. represents the standard error of the estimated parameter coefficient, SMC stands for squared multiple correlations, CR signifies composite reliability, AVE denotes average variance extracted, Alpha represents Cronbach’s α.

### 4.3 Structural model test

**Figure 2:**
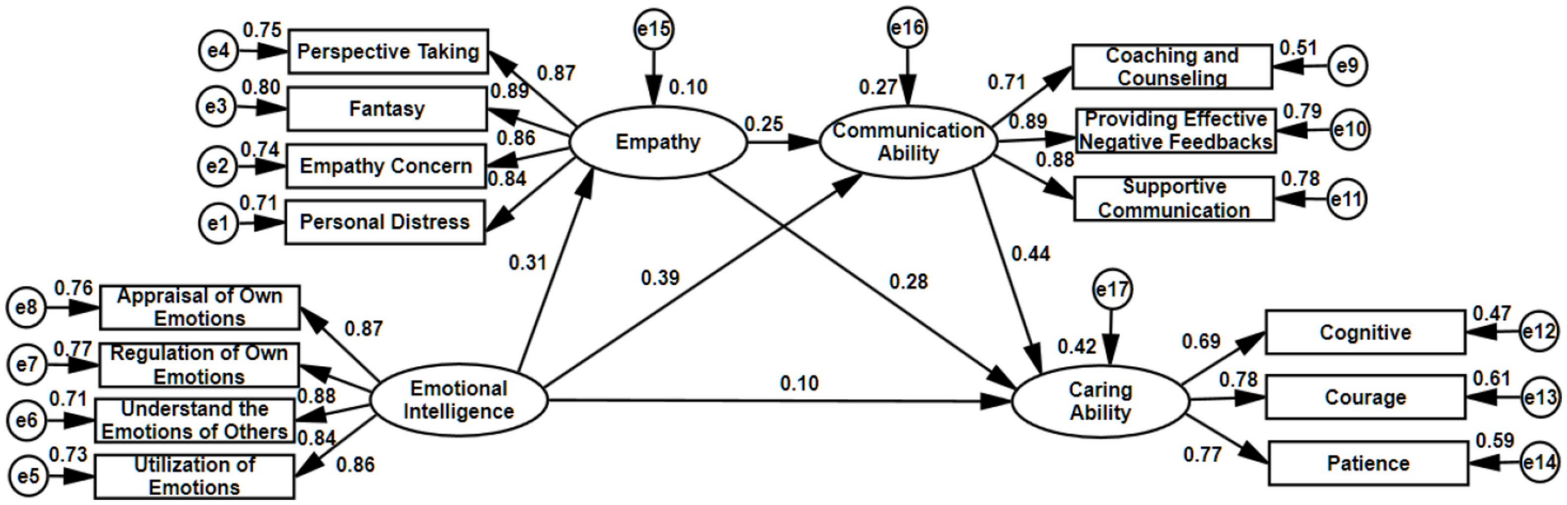
Structural equation modeling of hypothesized model. Standardized coefficients are shown.

SEM was developed (see Figure. 2), and the hypothesis model was subjected to fitting calculation through maximum likelihood estimation. According to the result of structural modeling, the proposed model fits the data well, as shown in Table 3. χ2 = 99.401, df = 71, χ2/df = 1.405, GFI = 0.988, AGFI = 0.982, CFI = 0.997, TLI = 0.997, SRMR = 0.019, RMSEA = 0.019.

**Table 3:** Model fitting index.

Fit indices	χ2 /df	GFI	AGFI	CFI	TLI	SRMR	RMSEA
Model value	1.400	0.988	0.982	0.997	0.997	0.019	0.019
Acceptable	<5	>0.90	>0.90	>0.90	>0.90	<0.08	<0.08
Excellent	<3	>0.95	>0.95	>0.95	>0.95	<0.06	<0.06

*χ*^2^/df = the chi-square degrees of freedom; GFI, goodness of fit index; AGFI, adjusted goodness of fit index; CFI, comparative normed fit index; TLI, Tucker-Lewis index; SRMR, standardized root mean square residual; RMSEA, root mean square error of approximation; Chi-square = 99.401, Degrees of freedom = 71.

The present study followed the recommendation of Baron and Kenny (1986), employing a causal steps approach to test the mediation’s first condition proposed in Hypotheses 1–6. The coefficients of correlation in Table 1 reflect positive associations. Table 4 reflects the results of the proposed model obtained after SEM analysis. Emotional intelligence substantially and positively influences caring ability (standardized estimate =0.385, *p*<0.001), empathy (standardized estimate =0.312, *p*<0.001), and communication ability (standardized estimate =0.464, *p*<0.001). Empathy substantially and positively influences caring ability (standardized estimate =0.470, *p*<0.001) and communication ability (standardized estimate =0.371, *p*<0.001). There was a significantly positive association between caring ability and communication ability (standardized estimate =0.584, *p*<0.001). Hypotheses 1–6 were thus supported.

**Table 4:** Study Hypothesis Test and Path Coefficient Values (N = 1,165).

Research hypothesis		Estimate	Standardized estimate	C.R.	*p*	Test Results
H1	Emotional intelligence→Caring ability	0.498	0.385	10.926	^***^	Supported
H2	Emotional intelligence→Empathy	0.147	0.312	10.007	^***^	Supported
H3	Empathy→Caring ability	1.281	0.470	13.159	^***^	Supported
H4	Emotional intelligence→Communication ability	0.092	0.464	13.994	^***^	Supported
H5	Communication ability→Caring ability	3.802	0.584	14.741	^***^	Supported
H6	Empathy→Communication ability	0.157	0.371	11.318	^***^	Supported

In the present study, bias-corrected percentile and percentile bootstrapping were conducted to examine the indirect influence of the dependent variable by using mediators. Table 5 manifests that 0 is excluded from both upper and lower ranges of the Percentile and Bias-corrected, and Z > 1.96, which suggests a significant mediating effect. The bootstrap test results validate that empathy played a crucial role in positively mediating the relationship between emotional intelligence and caring ability (standardized indirect effect =0.121), and communication ability mediated the correlation between emotional intelligence and caring ability (standardized indirect effect =0.169). Additionally, the examination also identified that empathy and communication ability played a crucial mediating role between caring ability and emotional intelligence (standardized indirect effect =0.034). The total indirect effect of the model accounted for 77.14% (0.324/0.420). As such, empathy and communication ability were partial mediators of emotional intelligence and caring ability, and were significant mediating factors. Hypotheses 7–9 were supported. Table 5 also shows that communication ability has a great medicating influence on caring ability and emotional intelligence was greater compared with empathy, but the difference was not statistically significant (z = 1.531).

**Table 5:** Standardized indirect and total effects of the hypothesized model.

SIE		Point estimation	Product of coefficient		Bias corrected 95%CI		Percentile 95%CI	
			SE	Z	Lower	Upper	Lower	Upper
H7	EI → IR → CA	0.121	0.019	6.368	0.087	0.163	0.085	0.159
H8	EI → SC → CA	0.169	0.022	7.682	0.129	0.214	0.127	0.212
H9	EI → IR → SC → CA	0.034	0.008	4.250	0.021	0.053	0.019	0.051
	SC minus IRI	0.049	0.032	1.531	0.108	0.017	0.110	0.016
	Total Indirect Effect	0.324	0.031	10.452	0.268	0.390	0.265	0.386
	Total Effect	0.420	0.032	13.125	0.359	0.485	0.357	0.482

Standardized estimating of 5,000 bootstrap sample. EI, Emotional Intelligence; IR = Empathy; SC, Communication ability; CA, Caring Ability.

## 5 Discussion

The interactions between empathy, communication, emotional intelligence, and caring ability was examined. The results show that the higher levels of former three elements enable nursing student to develop better caring ability. Additionally, it is observed that empathy and communication ability can indirectly affect how emotional intelligence influences caring ability. The influence mechanism of humanistic care has been explored, providing reference for cultivating the caring ability.

### 5.1 The relationship between emotional intelligence and caring ability

This paper confirms that emotional intelligence substantially and positively influences caring ability, supporting hypothesis 1 (Lina et al., 2022; Ma et al., 2022). High-emotional-intelligence groups tend to possess greater sensitivity to their own emotional status, improved self-regulation of emotions, and enhanced understanding of others’ emotions, all of which can lead to greater awareness, positive attitudes, and caring behavior toward others. Thus, emotional intelligence is positively related to higher levels of caring ability. To promote patient recovery, nursing students should be able to understand others, and establish harmonious interpersonal relationship with patients. Several studies (Prado-Gascó et al., 2019; Foji et al., 2020) have shown that emotional intelligence does not exist with freshmen, but requires reinforcement through postnatal learning. According to Freshwater and Stickley (2004), it is important to incorporate emotional intelligence competencies into the nursing curriculum because it is an essential part of nursing practice apart from physical tasks. This requires nursing educators to invest more effort in the training and guidance of emotional intelligence through targeted activities such as emotional experience activities, emotional intelligence lectures and team psychological counseling, so that nursing students can learn the knowledge and skills to recognize and regulate emotions, accurately identify and understand the emotional changes of patients and their families, and improve nursing students’ caring ability.

### 5.2 The mediating and chain mediating effects of empathy and communication ability

Firstly, empathy directly and positively impacts caring ability, and also plays a mediating role in the association between emotional intelligence and caring ability, hypothesis 2, hypothesis 3 and hypothesis 7 are supported, similar to previous research (Lina et al., 2022; Xiang et al., 2022; Wan et al., 2023). Empathy refers to the ability to comprehend and resonate with others’ emotions as if they were one’s own. Nursing students possessing better emotional intelligence have a stronger potential to perceive and accept others and are more empathetic. Such ability helps nursing students to comprehend patients’ emotions and needs, enabling them to provide personalized care from the patient’s perspective. Therefore, emotional intelligence facilitates the integration of cognitive, emotional, and motivational factors for the implementation of humanistic care. To enhance caring abilitiy of nursing students, teaching reforms are necessary. Several nursing educators have combined experience learning with the development of empathy skills, providing nursing students with the opportunity to learn from real-life experiences that elicit strong emotions like joy, pain, fear, and disappointment (Freshwater and Stickley, 2004; Wang et al., 2020). Moreover, to enhance their ability to empathize, nursing students should focus on improving their understanding of empathy and overcoming any self-centeredness in their daily practice, which can achieved by learning to see situations from the perspective of others and cultivating a sensitivity to the needs of others.

Secondly, communication ability positively influences caring ability, and can also mediate the effect of emotional intelligence on caring ability, supporting hypothesis 4, hypothesis 5, and hypothesis 8. The findings are supported by previous research (Raeissi et al., 2019; Li et al., 2021b, 2022). Moreover, This study also identified the role of empathy in improving communication skills, and hypothesis 6 is supported (Cannity et al., 2021; Chen et al., 2021). Nursing students who have high emotional intelligence are not only good at managing their emotions, but also good at using and regulating emotions (Petrovici and Dobrescu, 2014). In communication with others, they are better able to detect emotional changes in others during communication, enabling them to respond appropriately and maintain positive relationships with patients. Nurses’ care, love and respect for patients and their families are demonstrated through communication. The establishment of harmonious interpersonal relationships is promoted by high-quality and effective interpersonal communication, and the coordination of interpersonal relationships is a prerequisite for nursing humanistic care practice. Communication ability is the foundation of humanistic care, which requires nursing educators to strengthen the training of communication skills, ability and attitude of nursing students, and adopt standardized patient, role play, case analysis, clinical scenario simulation and other teaching approaches to enhance nursing students’ interpersonal communication ability. Meanwhile, nursing students should also consciously participate in social practice, through participating in school activities, student association activities and off-campus social activities to practice their interpersonal communication skills, thereby laying a foundation for the development of caring ability.

Thirdly, the present study finds that communication ability and empathy play a chain mediation in the influence caring ability. Specifically, these mediators accounted for 77.14% of the total effect, supporting hypothesis 9. Strong emotional intelligence is beneficial in improving nursing students’ empathy ability, and higher empathy ability is conducive to harmonious coexistence between both parties, further enhancing their caring ability. Empathy and interpersonal communication are significant mediators of emotional intelligence and humanistic care. Before implementing humanistic care, clinical practitioners should understand each other’s health problems and needs. Hence, it is essential for nursing students to possess the capability of comprehending patients’ emotional state and identifying their psychological needs to develop individualized and empathetic nursing interventions based on patients’ physiological, psychological, and social demands. Effective communication plays a crucial role in implementing and providing humanistic care, which is the basis and necessity of high quality nursing. Therefore, nursing managers and educators should invest more effort in fostering nursing students’ communication ability, emotional intelligence, and empathy, so as to strengthen the nursing abilities.

## 6 Limitations

Some limitations of the research need to be summarized. First, the research was conducted only in the junior college. Therefore, researchers need to consider this situation in the summary of results. Second, the adoption of a self-evaluation approach may lead to bias in the research results. In this sense, future studies should deeply explore nurses and nursing students from different regions and different educational backgrounds to improve the generalizability of the conclusions. A potential avenue for future research could be incorporating qualitative methods to complement the quantitative findings of the present study. Additionally, researchers could investigate other potential mediators and factors that potentially influence the development of nursing students’ caring abilities, beyond just empathy and communication skills. Through such research, more evidence can be gathered to inform effective approaches for cultivating nurses’ caring abilities.

## 7 Conclusion

This study presents its observations that emotional intelligence, empathy, and communication ability influence the humanistic caring ability, both directly and indirectly. Conclusions are as follows: (1) Emotional intelligence, empathy, and communication ability were found to positively affect nursing students’ caring ability. (2) Communication ability and empathy play an important role in mediating the association between emotional intelligence and caring ability, respectively. (3) Empathy and communication ability played a chain-mediating role between emotional intelligence and care ability.

This study confirmed the existing research results and proposed new findings, providing a new perspective for improving students’ caring ability. It is essential to continuously improve nursing students’ caring ability. From the perspective of nursing educators, they need to develop targeted approaches to help the nursing student improve their emotional intelligence, empathy, and communication skills, further enhancing their caring ability. It suggests improving students’ humanistic caring ability by integrating courses related to “empathy,” “emotional intelligence,” and “communication ability” into the existing curriculum. Moreover, it is recommended that nursing educators collaborate more closely between academic and clinical settings to develop effective educational interventions, such as utilizing standardized patient application, role play, reflective diary, and clinical situation performance, to improve and maintain nursing students’ empathy and communication abilities and ultimately enhance their humanistic care abilities.

## Data availability statement

The original contributions presented in the study are included in the article/supplementary material, further inquiries can be directed to the corresponding author.

## Ethics statement

The studies involving humans were approved by Ethics Committee of Huangshan Vocational and Technical College. The studies were conducted in accordance with the local legislation and institutional requirements. The participants provided their written informed consent to participate in this study.

## Author contributions

YY: Data curation, Investigation, Methodology, Project administration, Software, Supervision, Validation, Writing – original draft, Writing – review & editing. CW: Data curation, Formal analysis, Software, Validation, Visualization, Writing – review & editing.

## References

[ref1] Anderson J. C. Gerbing D. W. (1888). Structural equation modeling in practice: a review and recommended two-step approach. Psychol. Bull. 103, 411–423. doi: 10.1037/0033-2909.103.3.411

[ref2] Aupia A. Lee T. T. Liu C. Y. Wu S. F. V. Mills M. E. (2018). Caring behavior perceived by nurses, patients and nursing students in Indonesia. J. Prof. Nurs. 34, 314–319. doi: 10.1016/j.profnurs.2017.11.013, PMID: 30055686

[ref3] Baron R. M. Kenny D. A. (1986). The moderator-mediator variable distinction in social psychological research: conceptual, strategic, and statistical considerations. J. Pers. Soc. Psychol. 51, 1173–1182. doi: 10.1037/0022-3514.51.6.1173, PMID: 3806354

[ref4] Cannity K. M. Banerjee S. C. Hichenberg S. Leon-Nastasi A. D. Howell F. Coyle N.. (2021). Acceptability and efficacy of a communication skills training for nursing students: building empathy and discussing complex situations. Nurse Educ. Pract. 50:102928. doi: 10.1016/j.nepr.2020.102928, PMID: 33310509 PMC10332407

[ref5] Chen H. Liu C. Cao X. Hong B. Huang D. H. Liu C. Y.. (2021). Effects of loving-kindness meditation on doctors’ mindfulness, empathy, and communication skills. Int. J. Environ. Res. Public Health 18:4033. doi: 10.3390/ijerph18084033, PMID: 33921271 PMC8069630

[ref6] Cheng L. Liu Y. L. Ke Y. Y. Wang W. R. (2016). Comparison of caring ability between Chinese and American nursing students. West. J. Nurs. Res. 39, 290–304. doi: 10.1177/0193945916656613, PMID: 27378729

[ref7] Foji S. Vejdani M. Salehiniya H. Khosrorad R. (2020). The effect of emotional intelligence training on general health promotion among nurse. J. Educ. Health Promot. 9:4. doi: 10.4103/jehp.jehp_134_19, PMID: 32154299 PMC7032022

[ref8] Fornell C. Larcker D. F. (1981). Evaluating structural equation models with unobservable variables and measurement error. J. Mark. Res. 18, 39–50. doi: 10.2307/3151312

[ref9] Freshwater D. Stickley T. (2004). The heart of the art: emotional intelligence in nurse education. Nurs. Inq. 11, 91–98. doi: 10.1111/j.1440-1800.2004.00198.x, PMID: 15154888

[ref11] Giménez-Espert M. D. C. Prado-Gascó V. J. Valero-Moreno S. (2019). Impact of work aspects on communication, emotional intelligence and empathy in nursing. Rev. Lat. Am. Enfermagem 27:e3118. doi: 10.1590/1518-8345.2933.3118 PMC5738859 29236842

[ref12] Guan R. Y. Qian M. Y. (2014). Reliabilities and validities of interpersonal reactivity index among nursing students. Chin. J. Clin. Psych. 22, 493–495.

[ref13] Hair J. F. Black W. C. Babin B. J. Anderson R. E. Tatham R. L. (2006). Multivariate data analysis (6th). New Jersey, USA: Prentice-Hall.

[ref14] Hu L. T. Bentler P. M. (1999). Cutoff criteria for fit indexes in covariance structure analysis: conventional criteria versus new alternatives. Struct. Equ. Model. Multidiscip. J. 6, 1–55. doi: 10.1080/10705519909540118

[ref15] Hu S. Chen J. Jiang R. Hu H. Hu Z. Gao X.. (2022). Caring ability of nursing students pre- and post-internship: a longitudinal study. BMC Nurs. 21, 1–7. doi: 10.1186/s12912-022-00921-2 35644615 PMC9150307

[ref16] Ioannidou F. Konstantikaki V. (2008). Empathy and emotional intelligence: what is it really about? Int. J. Caring Sci. 1, 118–123.

[ref17] Jian S. Ya M. Qian Z. Meihua Y. Cao X. Dela Rosa R. D. (2022). Research progress on humanistic care ability and influencing factors of intern nursing students. Eur. Rev. Med. Pharmacol. Sci. 26, 8637–8643. doi: 10.26355/eurrev_202212_30534, PMID: 36524483

[ref18] Jöreskog K. G. Sörbom D. (1982). Recent developments in structural equation Modeling. J. Mark. Res. 19, 404–416. doi: 10.1177/002224378201900402

[ref19] Korkmaz Doğdu A. Aktaş K. Dursun Ergezen F. Bozkurt S. A. Ergezen Y. Kol E. (2022). The empathy level and caring behaviors perceptions of nursing students: a cross-sectional and correlational study. Perspect. Psychiatr. Care 58, 2653–2663. doi: 10.1111/ppc.13106, PMID: 35524462

[ref20] Kuo Y. L. Lee J. T. Yeh M. Y. (2021). Intergenerational narrative learning to bridge the generation gap in humanistic care nursing education. Healthcare 9:1291. doi: 10.3390/healthcare9101291, PMID: 34682971 PMC8535847

[ref21] Létourneau D. Goudreau J. Cara C. (2021). Humanistic caring, a nursing competency: modelling a metamorphosis from students to accomplished nurses. Scand. J. Caring Sci. 35, 196–207. doi: 10.1111/scs.12834, PMID: 32141649

[ref22] Létourneau D. Goudreau J. Cara C. (2022). Nursing students and nurses’ recommendations aiming at improving the development of the humanistic caring competency. Can. J. Nurs. Res. 54, 292–303. doi: 10.1177/08445621211048987, PMID: 34704493 PMC9379384

[ref23] Li X. Chang H. Zhang Q. Yang J. Liu R. Song Y. (2021a). Relationship between emotional intelligence and job well-being in Chinese clinical nurses: multiple mediating effects of empathy and communication satisfaction. BMC Nurs. 20, 144–110. doi: 10.1186/s12912-021-00658-4, PMID: 34389005 PMC8361242

[ref24] Li X. Fang X. Wang L. Geng X. Chang H. (2021b). Relationship between emotional intelligence and job well-being in Chinese registered nurses: mediating effect of communication satisfaction. Nurs. Open 8, 1778–1787. doi: 10.1002/nop2.820, PMID: 33656788 PMC8186701

[ref25] Li T. Jiang T. Shi G. Song C. Shi T. (2022). Correlation between self-awareness, communication ability and caring ability of undergraduate nursing students/a cross-sectional study. Nurse Educ. Today 116:105450. doi: 10.1016/j.nedt.2022.105450, PMID: 35797836

[ref26] Lina M. Qin G. Yang L. (2022). Mediating effects of emotional intelligence on the relationship between empathy and humanistic care ability in nursing students: a cross-sectional descriptive study. Medicine 101, e31673–e31675. doi: 10.1097/MD.0000000000031673, PMID: 36401377 PMC9678621

[ref27] Liu H. Zhang L. Yan J. Huang H. Yi Q. Peng L. (2023). The relationship between social support, empathy, self-efficacy, and humanistic practice ability among clinical nurses in China: a structural equation model. J. Nurs. Manag. 2023, 1–9. doi: 10.1155/2023/1378278 PMC11919114 40225687

[ref28] Ma J. Peng W. Pan J. Ma J. Peng W. Pan J. (2022). Investigation into the correlation between humanistic care ability and emotional intelligence of hospital staff. BMC Health Serv. Res. 22, 839–812. doi: 10.1186/s12913-022-08227-4, PMID: 35773661 PMC9244559

[ref29] Mayer J. D. Salovey P. Caruso D. R. (2008). Emotional intelligence: new ability or eclectic traits? Am. Psychol. 63, 503–517. doi: 10.1037/0003-066X.63.6.503 18793038

[ref30] Petrovici A. Dobrescu T. (2014). The role of emotional intelligence in building interpersonal communication skills. Procedia Soc. Behav. Sci. 116, 1405–1410. doi: 10.1016/j.sbspro.2014.01.406

[ref31] Prado-Gascó V. J. Giménez-Espert M. D. C. Valero-Moreno S. (2019). The influence of nurse education and training on communication, emotional intelligence, and empathy. Rev. Esc. Enferm. U.S.P. 53:e03465. doi: 10.1590/S1980-220X2018015903465 31365723

[ref32] Raeissi P. Zandian H. Mirzarahimy T. Delavari S. Moghadam T. Z. Rahimi G. (2019). Relationship between communication skills and emotional intelligence among nurses. Nurs. Manag. 26, 31–35. doi: 10.7748/nm.2019.e1820 31468761

[ref33] Rogers C. R. (1981). The foundations of the person-Centered approach. Dialect. Hum. 8, 5–16. doi: 10.5840/dialecticshumanism19818123

[ref35] Silva J. T. N. Toledo Júnior A. (2021). Association between emotional intelligence and empathy among medical students: a single center cross-sectional study, Brazil, 2019. Revista Brasileira de Educação Médica 45:e046. doi: 10.1590/1981-5271v45.1-20200053.ing

[ref36] Wan S. Lin S. Yirimuwen L. S. Qin G. (2023). The relationship between teacher-student relationship and adolescent emotional intelligence: a chain-mediated mediation model of openness and empathy. Psychol. Res. Behav. Manag. 16, 1343–1354. doi: 10.2147/prbm.S399824, PMID: 37114247 PMC10126722

[ref37] Wang C. (2002). Emotional intelligence, general self-efficacy and coping style of delinquent teenagers. Chin. J. Mental Health 16, 566–567. doi: 10.3321/j.issn:1000-6729.2002.08.022

[ref38] Wang Y. Zhang Y. Liu M. Zhou L. Zhang J. Tao H.. (2020). Research on the formation of humanistic care ability in nursing students: a structural equation approach. Nurse Educ. Today 86:104315. doi: 10.1016/j.nedt.2019.104315, PMID: 31896034

[ref39] Wessel J. Larin H. Benson G. Brown B. Ploeg J. Williams R. (2008). Emotional-social intelligence in health science students and its relation to leadership, caring and moral judgment. Internet J. Allied Health Sci. Pract. 6, 1–9. doi: 10.46743/1540-580X/2008.1180

[ref40] Xiang D. Qin G. Zheng X. (2022). The influence of student-teacher relationship on school-age Children’s empathy: the mediating role of emotional intelligence. Psychol. Res. Behav. Manag. 15, 2735–2744. doi: 10.2147/prbm.S380689, PMID: 36172541 PMC9511970

[ref41] Xie J. Ding S. Wang C. Liu A. (2013). An evaluation of nursing students' communication ability during practical clinical training. Nurse Educ. Today 33, 823–827. doi: 10.1016/j.nedt.2012.02.011 22417661

[ref42] Xu T. Wang Y. Wang R. Lamb K. V. Ren D. Dai G.. (2021). Predictors of caring ability and its dimensions among nurses in China: a cross-sectional study. Scand. J. Caring Sci. 35, 1226–1239. doi: 10.1111/scs.12941, PMID: 33615516

[ref43] Xue M. Sun H. Xue J. Zhou J. Qu J. Ji S.. (2023). Narrative medicine as a teaching strategy for nursing students to developing professionalism, empathy and humanistic caring ability: a randomized controlled trial. BMC Med. Educ. 23:38. doi: 10.1186/s12909-023-04026-5, PMID: 36653810 PMC9850682

[ref44] Zhong X. Liu X. Sheng Y. (2021). The effect of the humanistic care teaching model on nurse patient conflict and nurse turnover intention in a pediatric outpatient department: results of a randomized trial. Transl. Pediatr. 10, 2016–2023. doi: 10.21037/tp-21-214, PMID: 34584871 PMC8429853

